# An immune signature to predict the prognosis of *ATRX*-wildtype glioma patients and guide immune checkpoint blockade therapy

**DOI:** 10.18632/aging.205088

**Published:** 2023-10-06

**Authors:** Wenpeng Cao, Ping Sun, Shipeng Luo, Zhirui Zeng, Chaolun Xiao, Wenfeng Yu, Shan Lei

**Affiliations:** 1 Department of Anatomy, Key Laboratory of Human Brain bank for Functions and Diseases of Department of Education of Guizhou, Guizhou Medical University, Guiyang 550009, Guizhou, China; 2 Department of Neurosurgery, The Second People Hospital of Guiyang, Guiyang 550009, Guizhou, China; 3 Key Laboratory of Endemic and Ethnic Diseases, Ministry of Education, School of Basic Medicine, Guizhou Medical University, Guiyang 550009, Guizhou, China; 4 Key Laboratory of Medical Molecular Biology, School of Basic Medicine, Guizhou Medical University, Guiyang 550009, Guizhou, China; 5 Department of Physiology, School of Basic Medicine, Guizhou Medical University, Guiyang 550009, Guizhou, China

**Keywords:** immunity, signature, glioma, ATRX wildtype, immune checkpoint blockade therapy

## Abstract

Immune and stromal cells contribute to glioma progression by infiltrating the tumor microenvironment. We used clinical characteristics, RNA sequencing data and the ESTIMATE algorithm to obtain stromal and immune scores for alpha thalassemia retardation syndrome X-linked (*ATRX*)-mutation-type (*ATRX*-mt) and *ATRX*-wildtype (*ATRX*-wt) glioma tissues from The Cancer Genome Atlas. To identify specific immune biomarkers of glioma, we compared the gene expression profiles of *ATRX*-wt glioma tissues with high vs. low immune/stromal scores, and discovered 162 differentially expressed genes. The protein-protein interaction network based on these results contained 80 interacting genes, of which seven (*HOXA5*, *PTPN2*, *WT1*, *HOXD10*, *POSTN*, *ADAMDEC1* and *MYBPH*) were identified as key prognostic genes via LASSO and Cox regression analyses. A risk model constructed using the expression of these seven genes could predict survival for *ATRX*-wt glioma patients, but was ineffective for *ATRX*-mt patients. T cells and macrophages were more prevalent in low-risk than in high-risk glioma tissues. Immune checkpoint blockade treatment was highly beneficial for patients with low risk scores. High-risk gliomas were predicted to be more sensitive to rapamycin, dasatinib, 5-fluorouracil and gemcitabine. Thus, our model can be used for the diagnosis, prognostic prediction and treatment planning of *ATRX*-wt glioma patients.

## INTRODUCTION

Among primary malignant tumors of the nervous system, gliomas are the most common. In recent years, molecular technology has revealed some of the genetic and chromosomal changes associated with the occurrence, development and prognosis of gliomas [1]. Gliomas can be characterized by several pathological molecular changes, including telomerase reverse transcriptase promoter mutations, isocitrate dehydrogenase mutations, methylguanine methyltransferase promoter methylation, 1p/19q codeletion and alpha thalassemia retardation syndrome X-linked (*ATRX*) mutations [2].

At tandem repeat sequences in the genome, *ATRX* helps to prevent replication fork arrest, facilitate histone variant formation and prevent homologous recombination at telomeres [3]. Loss of *ATRX* causes epigenetic changes, including hypomethylation of repetitive elements such as telomeres [4]. *ATRX* mutation or loss is common in a variety of tumor types, including low-grade astrocytomas and secondary glioblastomas [5]. A bioinformatic analysis revealed that glioblastoma patients with *ATRX* loss had longer overall survival and greater benefit from temozolomide treatment than patients without this change [6]. However, it is unclear how the dysregulation of stromal and immune cell infiltration in *ATRX*-wildtype (*ATRX*-wt) glioma influences its development and progression. Therefore, identifying specific biomarkers of *ATRX*-wt glioma may facilitate the treatment of this disease.

The Cancer Gene Atlas (TCGA) is commonly used to identify tumor biomarkers in bioinformatic analyses [7]. Zhu et al. found that a nuclear translocation-associated gene signature combined with isocitrate dehydrogenase mutation status and 1p/19q codeletion status could improve prognostic prediction in glioma patients [8]. Feng et al. identified and validated an autophagy-associated signature to predict survival in low-grade gliomas [9]. In this study, we used TCGA data to construct a specific risk model that predicts the prognosis of *ATRX*-wt glioma patients and informs immune checkpoint blockade (ICB) therapy. Our results may provide new insights into the diagnosis and treatment of *ATRX*-wt gliomas.

## RESULTS

### High- and low-immune/stromal grouping of *ATRX*-mt and *ATRX*-wt glioma patients in TCGA

**Figure 1:**
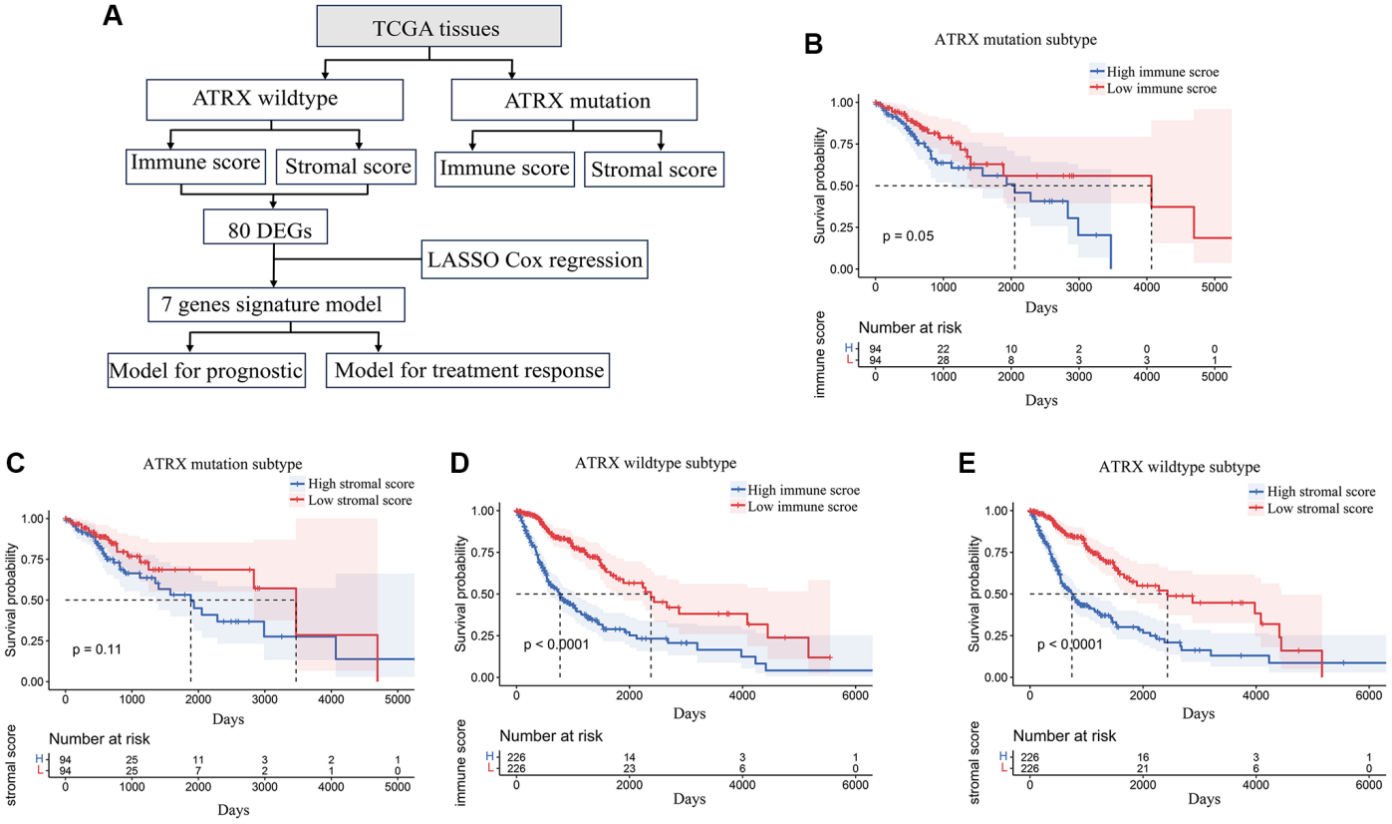
**Stromal and immune scores were associated with survival in *ATRX*-wt glioma patients.** (**A**) Flow chart of the analytical process in this study. (**B**) Kaplan-Meier survival analysis for *ATRX*-mt glioma patients in the high- and low-immune-score groups. (**C**) Kaplan-Meier survival analysis for *ATRX*-mt glioma patients in the high- and low-stromal-score groups. (**D**) Kaplan-Meier survival analysis for *ATRX*-wt glioma patients in the high- and low-immune-score groups. (**E**) Kaplan-Meier survival analysis for *ATRX*-wt glioma patients in the high- and low-stromal-score groups.

Our workflow to identify, test and validate prognostic and predictive biomarkers of glioma is shown in Figure 1A. The ESTIMATE algorithm was used to calculate immune and stromal scores for *ATRX*-mutation-type (*ATRX*-mt) and *ATRX*-wt glioma patients from TCGA, and the median scores were used to divide patients into high- and low-scoring groups. According to a Kaplan-Meier survival analysis, high vs. low immune/stromal scores did not significantly impact *ATRX*-mt glioma patient survival (Supplementary Table 1; Figure 1B, 1C). However, high immune and stromal scores were associated with poorer overall survival than low immune/stromal scores in *ATRX*-wt glioma patients (Figure 1D, 1E).

**Figure 2:**
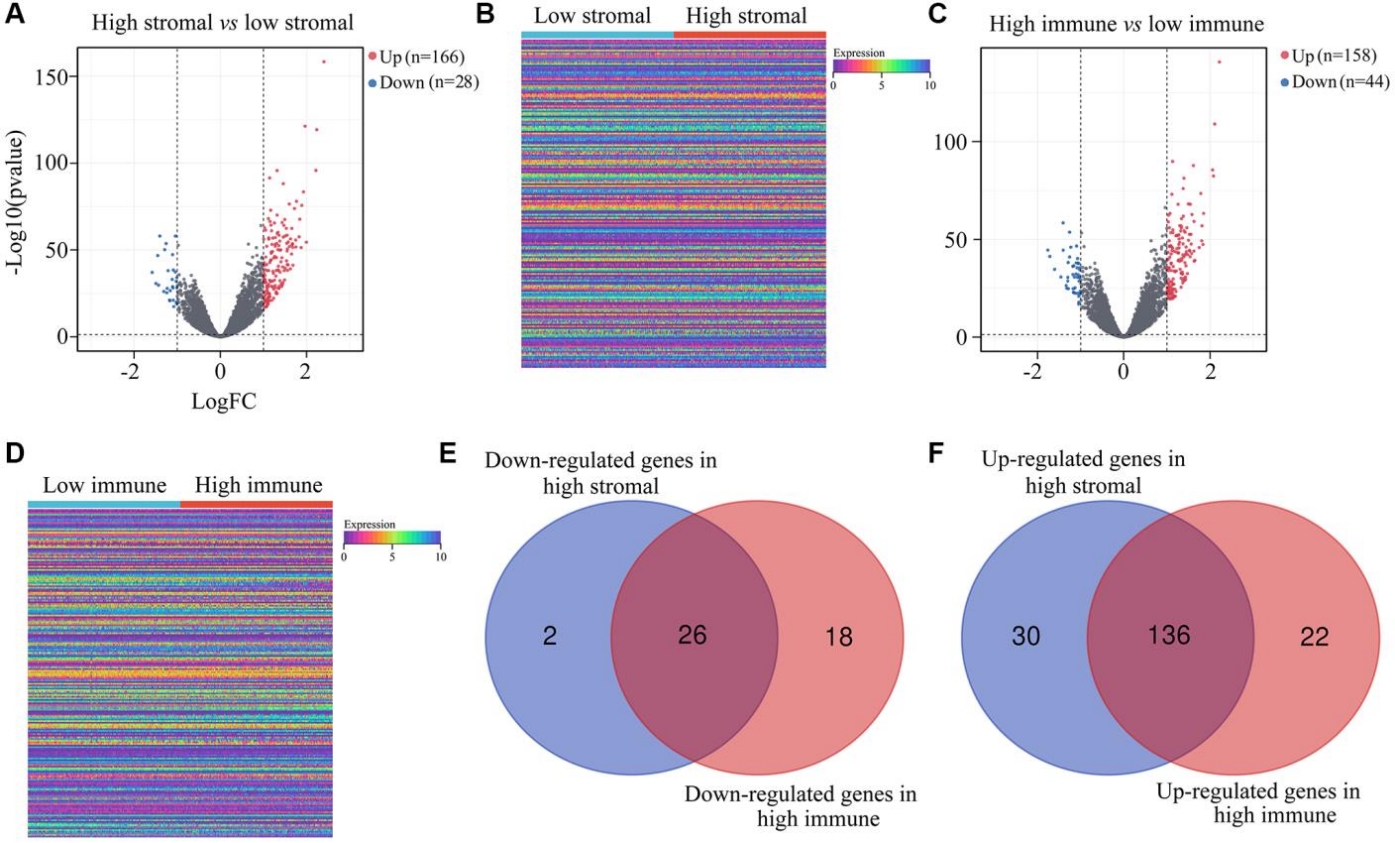
**DEGs in *ATRX*-wt glioma tissues with high vs. low stromal/immune scores.** (**A**) Volcano plot showing the DEGs between the high- and low-stromal-score groups of *ATRX*-wt glioma tissues. (**B**) Heat map showing the DEGs between the high- and low-stromal-score groups of *ATRX*-wt glioma tissues. (**C**) Volcano plot showing the DEGs between the high- and low-immune-score groups of *ATRX*-wt glioma tissues. (**D**) Heat map showing the DEGs between the high- and low-immune-score groups of *ATRX*-wt glioma tissues. (**E**) Overlapping downregulated genes between the high-stromal-score and high-immune-score groups of *ATRX*-wt glioma tissues. (**F**) Overlapping upregulated genes between the high-stromal-score and high-immune-score groups of *ATRX*-wt glioma tissues.

Next, we compared the gene expression profiles of *ATRX*-wt glioma patients with high and low immune/stromal scores. We found 166 upregulated and 28 downregulated genes in the high-stromal-score group compared with the low-stromal-score group (Figure 2A, 2B). In addition, we identified 158 upregulated and 44 downregulated genes in high-immune-score patients compared with low-immune-score patients (Figure 2C, 2D). When we analyzed the differentially expressed genes (DEGs) that overlapped between the high-stromal-score and high-immune-score groups of *ATRX*-wt glioma tissues, we found 136 upregulated and 26 downregulated genes (Supplementary Table 2; Figure 2E, 2F).

**Figure 3:**
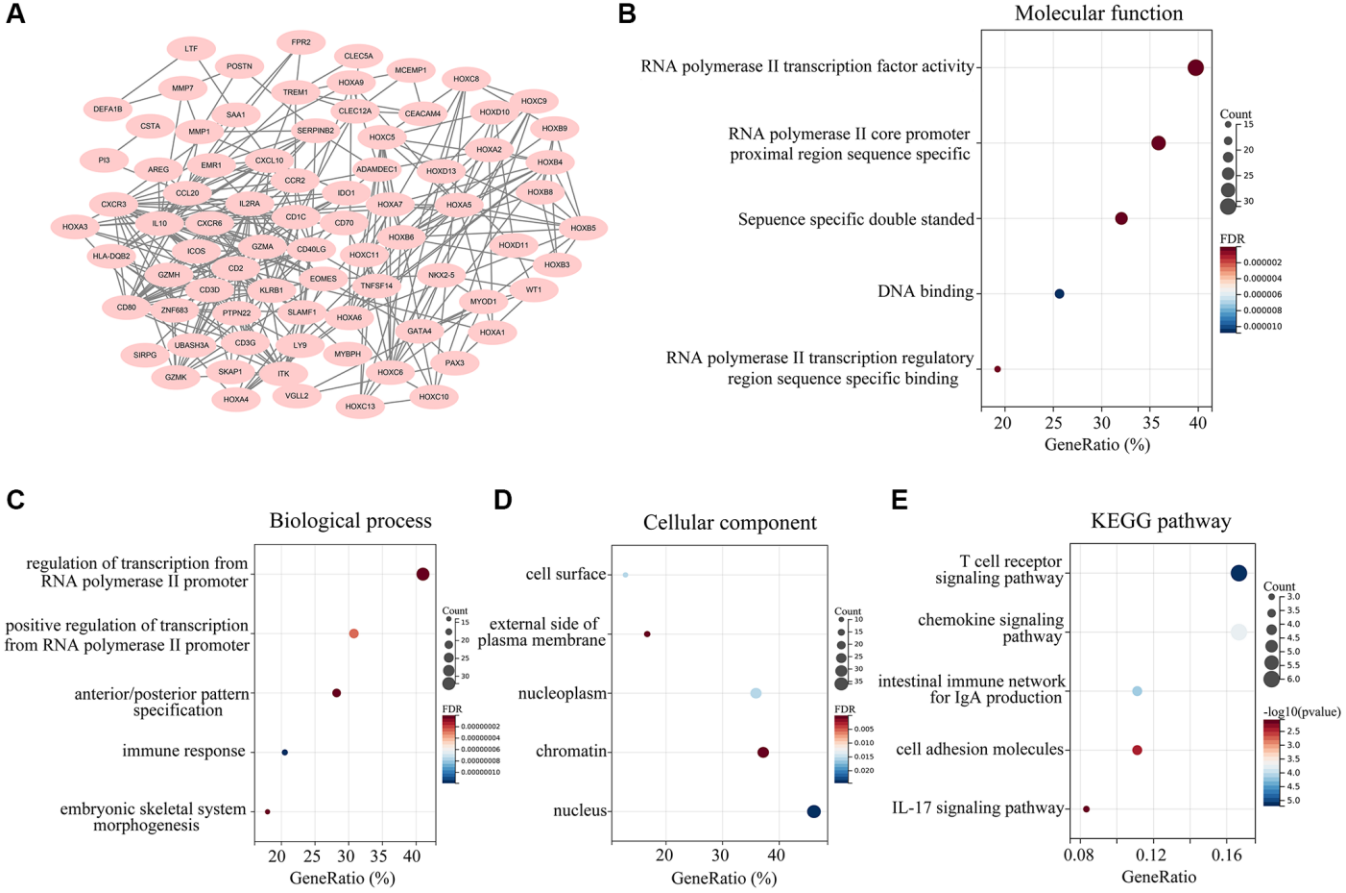
**Landscape of the 162 overlapping DEGs.** (**A**) A PPI network was constructed using the 162 overlapping DEGs, and isolated genes were removed. Genes in the PPI network were set as candidate hub genes. (**B**) Molecular Function analysis of candidate hub genes. (**C**) Biological Process analysis of candidate hub genes. (**D**) Cellular Component analysis of candidate hub genes. (**E**) KEGG analysis of candidate central genes.

We then generated a protein-protein interaction (PPI) network based on the overlapping DEGs in *ATRX*-wt glioma patients with high stromal and immune scores. The PPI network contained 80 interacting genes (Figure 3A). Candidate hub genes from this network were subjected to Gene Ontology analyses for Molecular Function, Biological Process and Cellular Component. The Molecular Function terms associated with the candidate hub genes were enriched in “RNA polymerase II transcription factor activity”, “RNA polymerase II core promoter proximal region sequence specific”, “sequence specific double stranded”, “DNA binding” and “RNA polymerase II transcription regulatory region sequence specific binding” (Figure 3B). In the Biological Process analysis, the candidate hub genes were found in “regulation of transcription from RNA polymerase II promoter”, “positive regulation of transcription from RNA polymerase II promoter”, “anterior/posterior pattern specification”, “immune response” and “embryonic skeletal system morphogenesis” (Figure 3C). The Cellular Component analysis was enriched in “cell surface”, “external side of plasma membrane”, “nucleoplasm”, “chromatin” and “nucleus” (Figure 3D). We also performed a Kyoto Encyclopedia of Genes and Genomes (KEGG) pathway analysis, which revealed that the candidate hub genes were involved in the “T cell receptor signaling pathway”, “chemokine signaling pathway”, “intestinal immune network for IgA production”, “cell adhesion molecules” and “IL-17 signaling pathway” (Figure 3E).

### Constructing the immune profiles of *ATRX*-wt glioma patients

**Figure 4:**
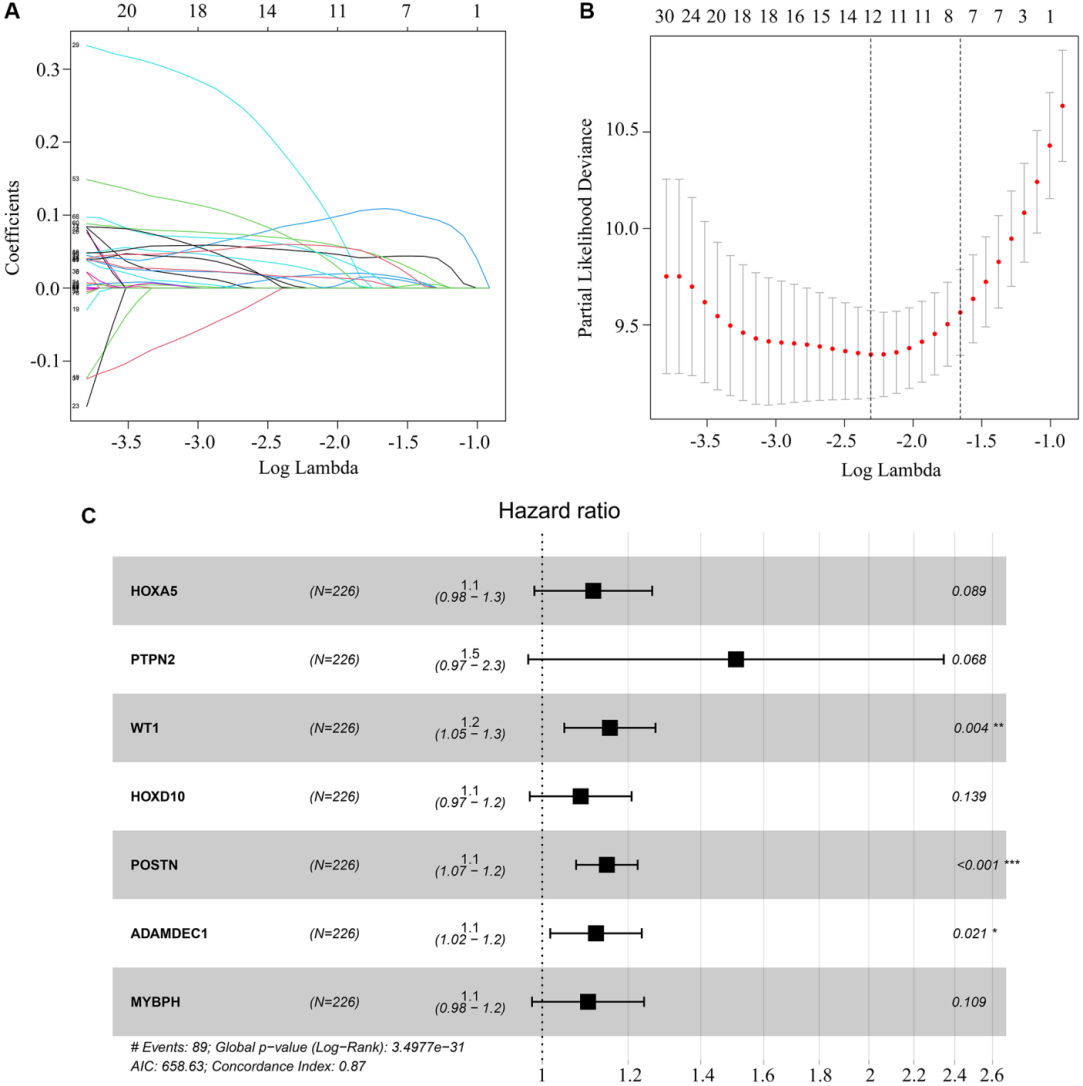
**Key genes selected for risk model construction.** (**A**, **B**) LASSO analysis of key genes associated with survival in *ATRX*-wt glioma patients. (**C**) Multivariate Cox regression analysis of *HOXA5*, *PTPN2*, *WT1*, *HOXD10*, *POSTN*, *ADAMDEC1* and *MYBPH*. These seven genes were used for risk model construction.

Next, we performed a univariate Cox regression analysis, which revealed that seven of the 80 aforementioned candidate genes were associated with survival in *ATRX*-wt glioma patients (Table 1). We subsequently conducted a LASSO analysis (Figure 4A, 4B) and a multivariate Cox analysis (Figure 4C), which identified homeobox A5 (*HOXA5*), protein tyrosine phosphatase non-receptor type 2 (*PTPN2I*, WT1 transcription factor (*WT1*), homeobox D10 (*HOXD10*), periostin (*POSTN*), ADAM like decysin 1 (*ADAMDEC1*) and myosin binding protein H (*MYBPH*) as independent predictors of survival in *ATRX*-wt glioma patients. These results were used to develop a risk model capable of predicting survival in *ATRX*-wt glioma patients. After computer optimization, and the following risk score expression was obtained: 0.11 × *HOXA5* expression + 0.41 × *PTPN2* expression + 0.14 × *WT1* expression + 0.08 *HOXD10* expression + 0.14 × *POSTN* expression + 0.11 × *ADAMDEC1* expression + 0.10 × MYBPH expression.

**Table 1:** The hazard rate of genes for glioma patients with *ATRX* wild type.

**ID**	**HR**	**HR.95L**	**HR.95H**	***p*-value**
IL10	1.63484617	1.412942779	1.891599604	3.99005E-11
CD2	1.611050947	1.433723056	1.810311372	1.09872E-15
CD80	1.7382012	1.499705417	2.01462459	2.10213E-13
CD40LG	1.8853422	1.569864227	2.264218236	1.14512E-11
CD3D	1.511986167	1.357567997	1.683968814	5.41101E-14
CXCR3	1.689515301	1.466473383	1.946480574	3.86946E-13
GZMA	1.533573702	1.368230012	1.718898341	2.03829E-13
IL2RA	1.420618668	1.310610237	1.539860855	1.37017E-17
CXCL10	1.444697765	1.327141928	1.572666485	1.9634E-17
KLRB1	1.424623253	1.252339783	1.620607633	7.38551E-08
CD1C	1.304011289	1.106812658	1.536344413	0.001508233
ITK	1.304789296	1.212904297	1.403635153	9.29442E-13
CCL20	1.528458983	1.379381296	1.693648355	5.37739E-16
EOMES	1.850243392	1.563956973	2.18893529	7.26351E-13
HOXA5	1.487580596	1.37233437	1.612504996	4.77108E-22
HOXB4	1.356548073	1.268566382	1.450631752	4.961E-19
HOXC5	1.355479717	1.247924496	1.47230483	5.56712E-13
HOXC6	1.398363699	1.299196088	1.505100771	4.09751E-19
CCR2	1.515505644	1.3446989	1.708008654	9.46906E-12
CD3G	1.97053978	1.675350136	2.317740596	2.56698E-16
CXCR6	1.632790049	1.434214112	1.858860069	1.25766E-13
GZMK	1.463623123	1.304237832	1.642486205	9.46095E-11
SLAMF1	1.819797159	1.547395713	2.140151786	4.58977E-13
HOXA6	1.662424064	1.494113871	1.849694204	1.03216E-20
HOXA7	1.345855826	1.259788559	1.437803106	1.26014E-18
HOXB5	1.358497599	1.260754795	1.46381813	8.83251E-16
HOXB6	1.326695547	1.210956287	1.453496788	1.28002E-09
ICOS	1.920980315	1.636669811	2.254679195	1.36592E-15
PTPN2	3.495063245	2.621384416	4.659929698	1.50998E-17
SERPINB2	1.066398994	0.956099402	1.189423205	0.248475874
HLA-DQB2	1.429799876	1.289408444	1.585477196	1.19892E-11
TREM1	1.396376391	1.288781934	1.512953412	3.31576E-16
GZMH	1.543481182	1.366668852	1.743168549	2.70548E-12
HOXA4	1.378181402	1.288339219	1.474288719	1.09728E-20
IDO1	1.394241778	1.279359915	1.519439612	3.58653E-14
EMR1	1.339801159	1.22325332	1.467453319	2.97954E-10
HOXA3	1.411515491	1.315087781	1.515013681	1.33904E-21
HOXC13	1.393680779	1.295503313	1.499298454	5.26971E-19
HOXC8	1.37465946	1.281366075	1.474745327	7.04381E-19
MMP1	1.306961437	1.19148689	1.433627355	1.41002E-08
SAA1	1.283128792	1.219917417	1.349615534	3.95869E-22
CLEC12A	1.644937171	1.464824546	1.847196174	4.04077E-17
HOXA2	1.394805161	1.299252034	1.497385716	3.92337E-20
HOXC10	1.341228692	1.261587965	1.425896927	5.45794E-21
HOXC11	1.403993146	1.302969576	1.512849409	5.28754E-19
HOXC9	1.436720784	1.331802034	1.549904985	7.54091E-21
ZNF688	0.874617662	0.624701343	1.224514824	0.435228095
GATA4	1.241703748	1.167891593	1.32018092	4.40649E-12
HOXA9	1.39322244	1.289617922	1.505150273	4.05828E-17
HOXB8	1.254756043	1.176395478	1.338336261	5.29039E-12
HOXB9	1.500349523	1.367894648	1.645630162	7.75209E-18
LY9	1.67557693	1.427630369	1.966586106	2.66302E-10
MYOD1	0.539023747	0.439750921	0.660707202	2.67179E-09
UBASH3A	1.567325372	1.384392666	1.774430682	1.27879E-12
WT1	1.335635519	1.234997982	1.44447381	4.46098E-13
CD70	1.432034962	1.309320043	1.566251234	3.96289E-15
HOXB3	1.345372479	1.266243377	1.429446454	8.59509E-22
HOXD10	1.379600692	1.288315904	1.477353546	3.17174E-20
MMP7	1.226784337	1.142975571	1.316738388	1.50129E-08
NKX2-5	1.444403467	1.34504005	1.551107252	4.9136E-24
PAX3	1.387602684	1.296537487	1.485064048	3.12651E-21
POSTN	1.278881874	1.215527437	1.345538405	2.32988E-21
SKAP2	1.895375614	1.597045547	2.249434103	2.52333E-13
CEACAM4	1.476404054	1.258849233	1.731556785	1.66575E-06
FPR2	1.490163867	1.326673876	1.67380122	1.72684E-11
HOXA1	1.697129654	1.52345432	1.890604152	7.77973E-22
HOXD11	1.38815997	1.295848113	1.487047814	9.49276E-21
C19orf59	1.546978426	1.394142512	1.716569311	2.02504E-16
TNFSF14	1.608910103	1.441944692	1.795208744	1.78337E-17
ADAMDEC1	1.389433804	1.281737235	1.506179459	1.35046E-15
AREG	1.38785747	1.242507701	1.550210397	6.36893E-09
CLEC5A	1.470656313	1.341530655	1.612210637	1.93109E-16
CSTA	1.556115415	1.37713758	1.758353864	1.3108E-12
DEFA1B	1.272612393	1.15436257	1.402975412	1.26672E-06
HOXD13	1.347733466	1.262427352	1.438803979	3.71652E-19
LTF	1.211324452	1.157232844	1.26794442	1.9472E-16
MYBPH	1.51820243	1.390962593	1.657081672	8.83792E-21
PI3	1.274011507	1.196291544	1.356780735	4.67323E-14
SIRPG	1.908303098	1.607189367	2.26583176	1.63867E-13
VGLL2	1.455690311	1.328701794	1.594815549	7.47498E-16

### Risk model validation for *ATRX*-wt glioma patients from TCGA

**Figure 5:**
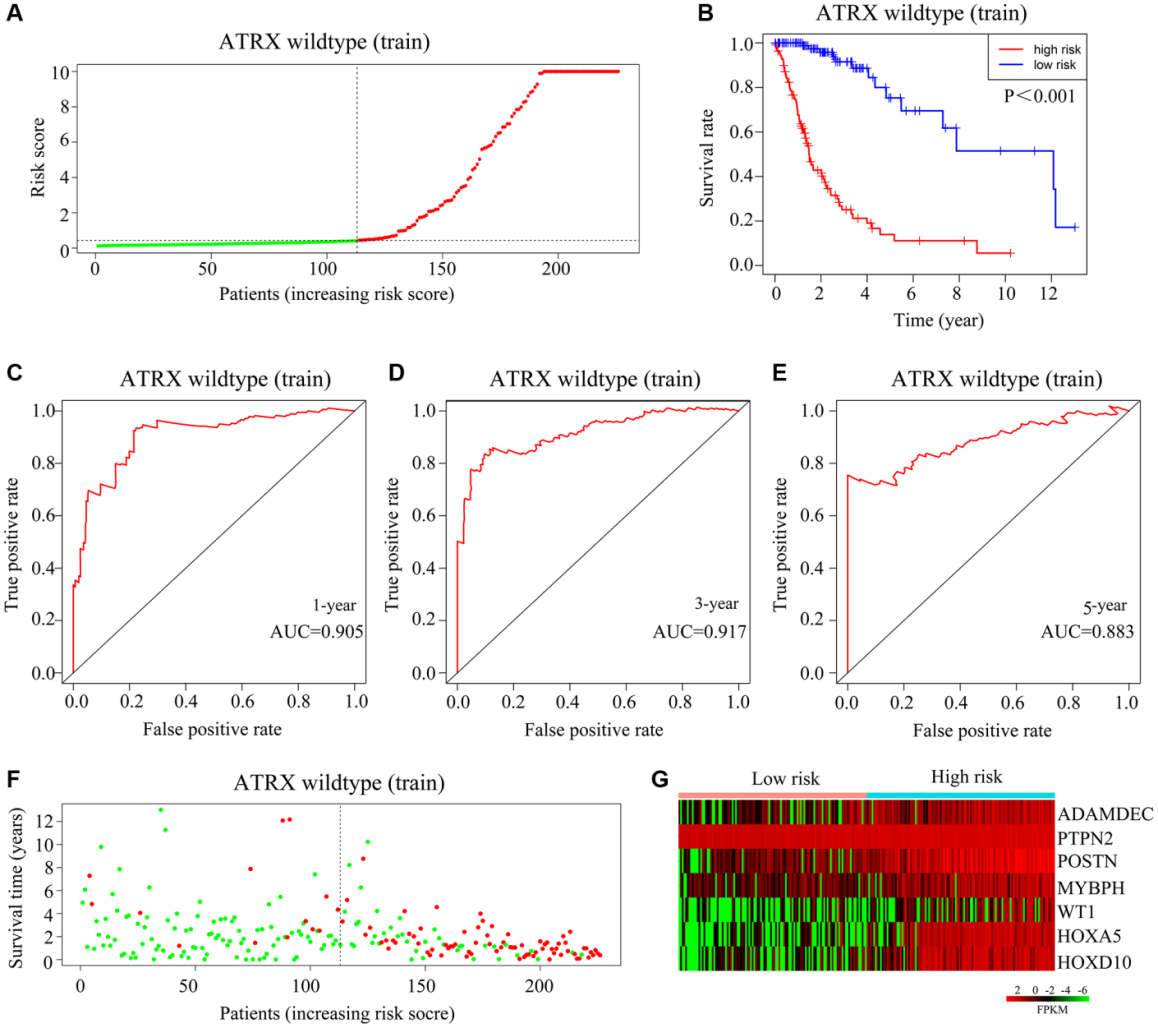
**Validation of the applicability of the risk model in the training cohort of *ATRX*-wt glioma patients.** (**A**) *ATRX*-wt glioma patients in the training cohort were divided into high- and low-risk-score groups based on the median risk score. (**B**) Survival differences between *ATRX*-wt glioma patients in the high- and low-risk-score groups in the training cohort. (**C**–**E**) Prognostic value of the risk model for the one-, three- and five-year survival of *ATRX*-wt glioma patients in the training cohort. (**F**) Deaths of *ATRX*-wt glioma patients in the high- and low-risk-score groups in the training cohort (green dots represent living cases; red dots represent dead cases). (**G**) Expression of *HOXA5*, *PTPN2*, *WT1*, *HOXD10*, *POSTN*, *ADAMDEC1* and *MYBPH* in glioma patients in the high- and low-risk-score groups in the training cohort.

Next, we randomized *ATRX*-wt glioma patients from TCGA into training and testing groups to evaluate the applicability of the risk model. The median risk score of *ATRX*-wt glioma patients in the training cohort was used to divide patients into high-risk and low-risk groups (Figure 5A). The overall survival rate was lower in the high-risk group than in the low-risk group (Figure 5B). The area under the curve (AUC) values for predicting survival after one, three and five years in *ATRX*-wt glioma patients from TCGA were 0.905, 0.917 and 0.883, respectively (Figure 5C–5E). The proportion of deaths among *ATRX*-wt glioma patients in the training cohort was higher in the high-risk-score group than in the low-risk-score group (Figure 5F). In glioma tissues from the high-risk group of the training cohort, *HOXA5*, *PTPN2*, *WT1*, *HOXD10*, *POSTN*, *ADAMDEC1* and *MYBPH* were highly expressed (Figure 5G).

**Figure 6:**
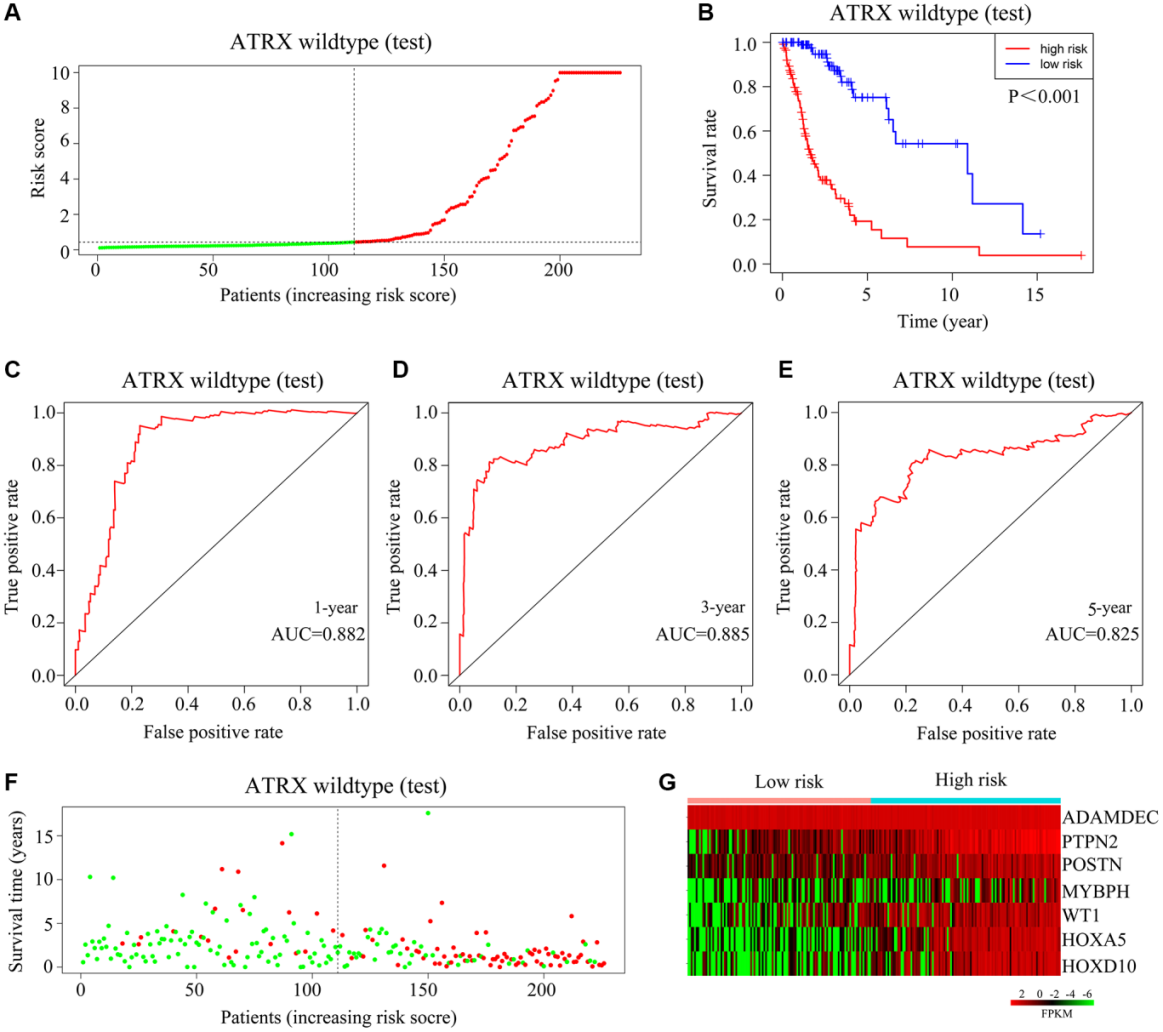
**Validation of the applicability of the risk model in the test cohort of *ATRX*-wt glioma patients.** (**A**) *ATRX*-wt glioma patients in the test cohort were divided into high- and low-risk-score groups based on the median risk score. (**B**) Survival differences between *ATRX*-wt glioma patients in the high- and low-risk-score groups in the test cohort. (**C**–**E**) Prognostic value of the risk model for one-, three- and five-year survival in *ATRX*-wt glioma patients in the test cohort. (**F**) Deaths of *ATRX*-wt glioma patients in the high- and low-score groups in the test cohort (green dots represent living cases; red dots represent dead cases). (**G**) Expression of *HOXA5*, *PTPN2*, *WT1*, *HOXD10*, *POSTN*, *ADAMDEC1* and *MYBPH* in glioma patients in the high- and low-risk-score groups of the test cohort.

We then validated the model using the test cohort. Again, *ATRX*-wt glioma patients were divided into high- and low-risk groups based on the median risk score (Figure 6A). Glioma patients with high-risk scores were more likely to die than those with low-risk scores (Figure 6B). The AUCs for predicting survival after one, three and five years in *ATRX*-wt glioma patients from the test cohort were 0.882, 0.885 and 0.825, respectively (Figure 6C–6E). The death rate of the high-risk group of glioma patients was higher in the test cohort than in the high-risk-score group than in the low-risk-score group (Figure 6F). In the test cohort, *HOXA5*, *PTPN2*, *WT1*, *HOXD10*, *POSTN*, *ADAMDEC1* and *MYBPH* levels were elevated in glioma tissues with high-risk scores (Figure 6G). Thus, our risk model exhibited significant prognostic value in *ATRX*-wt glioma patients.

### Exploring the applicability of the risk model for *ATRX*-mt glioma patients from TCGA

**Figure 7:**
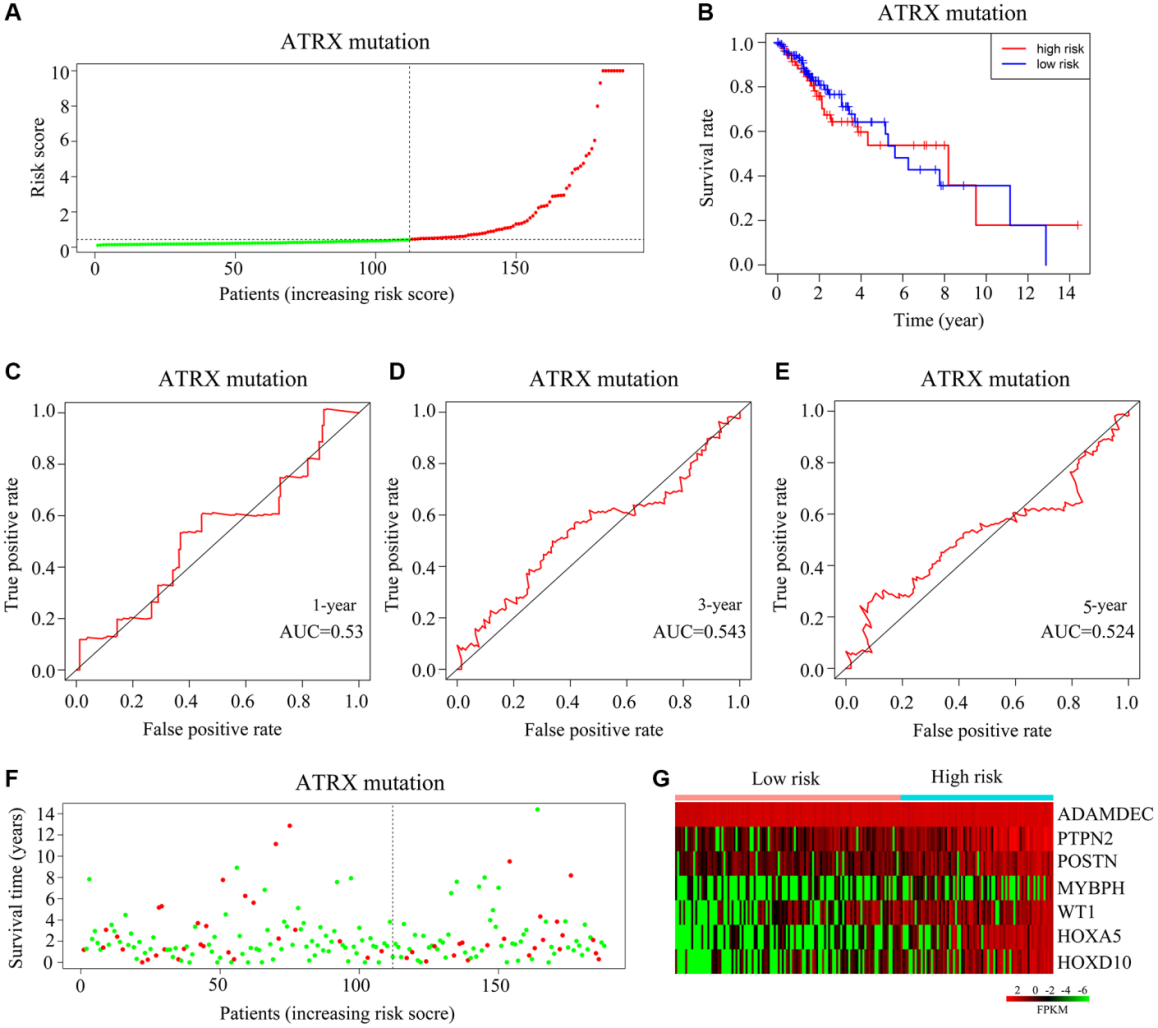
**Validation of the applicability of the risk model in *ATRX*-mt glioma patients from TCGA.** (**A**) *ATRX*-mt glioma patients from TCGA were divided into high- and low-risk-score groups based on the median risk score. (**B**) Survival differences between the high- and low-risk-score groups of *ATRX*-mt glioma patients from TCGA. (**C**–**E**) Prognostic value of the risk model for the one-, three- and five-year survival of *ATRX*-mt glioma patients from TCGA. (**F**) Deaths of *ATRX*-mt glioma patients from TCGA in the high- and low-risk-score groups (green dots represent living cases; red dots represent dead cases). (**G**) Expression of *HOXA5*, *PTPN2*, *WT1*, *HOXD10*, *POSTN*, *ADAMDEC1* and *MYBPH* in *ATRX*-mt glioma tissues from the high- and low-risk-score groups.

Next, we classified *ATRX*-mt patients from TCGA into high- and low-risk groups based on the median risk score (Figure 7A). High risk scores were associated with a lower survival rate than low risk scores (Figure 7B). However, the AUCs for predicting one-, three- and five-year survival among *ATRX*-mt glioma patients were 0.53, 0.543 and 0.524, respectively (Figure 7C–7E). Moreover, the death rates for *ATRX*-mt glioma patients did not differ significantly between the high- and low-risk groups (Figure 7F). Nevertheless, *HOXA5*, *PTPN2*, *WT1*, *HOXD10*, *POSTN*, *ADAMDEC1* and *MYBPH* were overexpressed in *ATRX*-mt glioma tissues with high-risk scores compared with low-risk scores (Figure 7G). These results demonstrated that the risk model constructed with *HOXA5*, *PTPN2*, *WT1*, *HOXD10*, *POSTN*, *ADAMDEC1* and *MYBPH* could not accurately predict survival in *ATRX*-mt glioma patients, although it could in *ATRX*-wt glioma patients.

### Immune characteristics as an independent prognostic factor for *ATRX*-wt glioma patients

**Figure 8:**
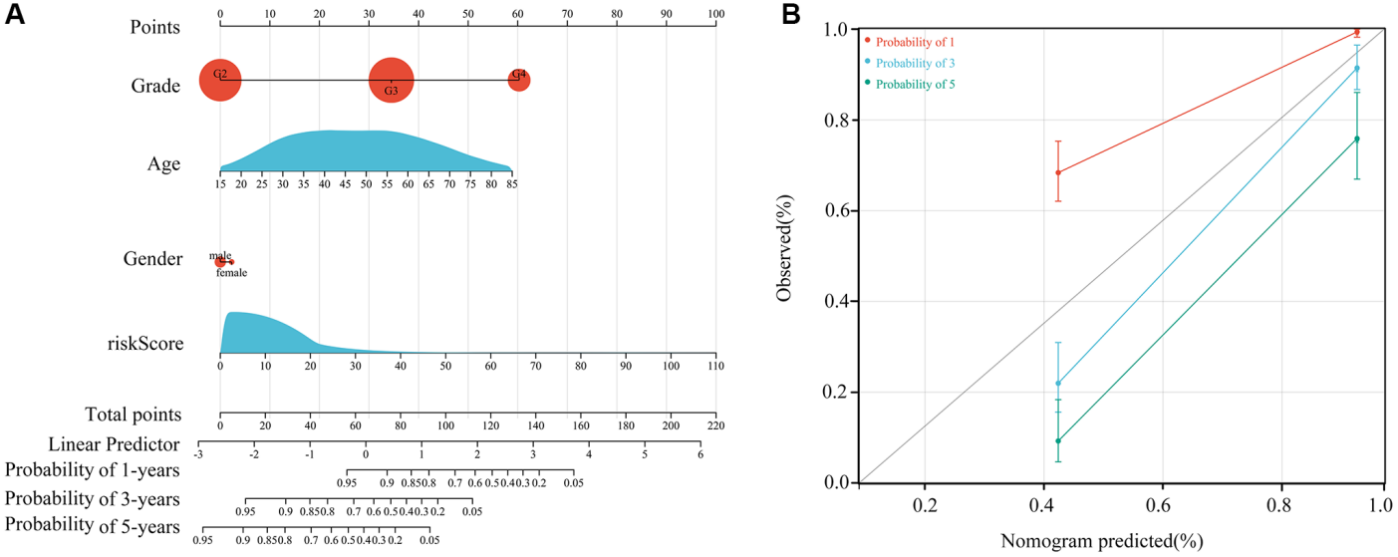
**Construction of the nomogram.** (**A**) Construction of a nomogram using age, sex, grade and risk score. (**B**) Nomogram in glioma patients with one-, three- and five-year survival.

Our multivariate Cox regression analysis indicated that the *HOXA5*-derived immune signature was independently associated with the outcomes of *ATRX*-wt glioma patients. We used this signature risk score and patients’ clinical characteristics to create a nomogram (Figure 8A), which had high prognostic value for survival at one, three and five years (Figure 8B).

### Expression of *HOXA5*, *PTPN2*, *WT1*, *HOXD10*, *POSTN*, *ADAMDEC1* and *MYBPH* in *ATRX*-wt glioma tissues

**Figure 9:**
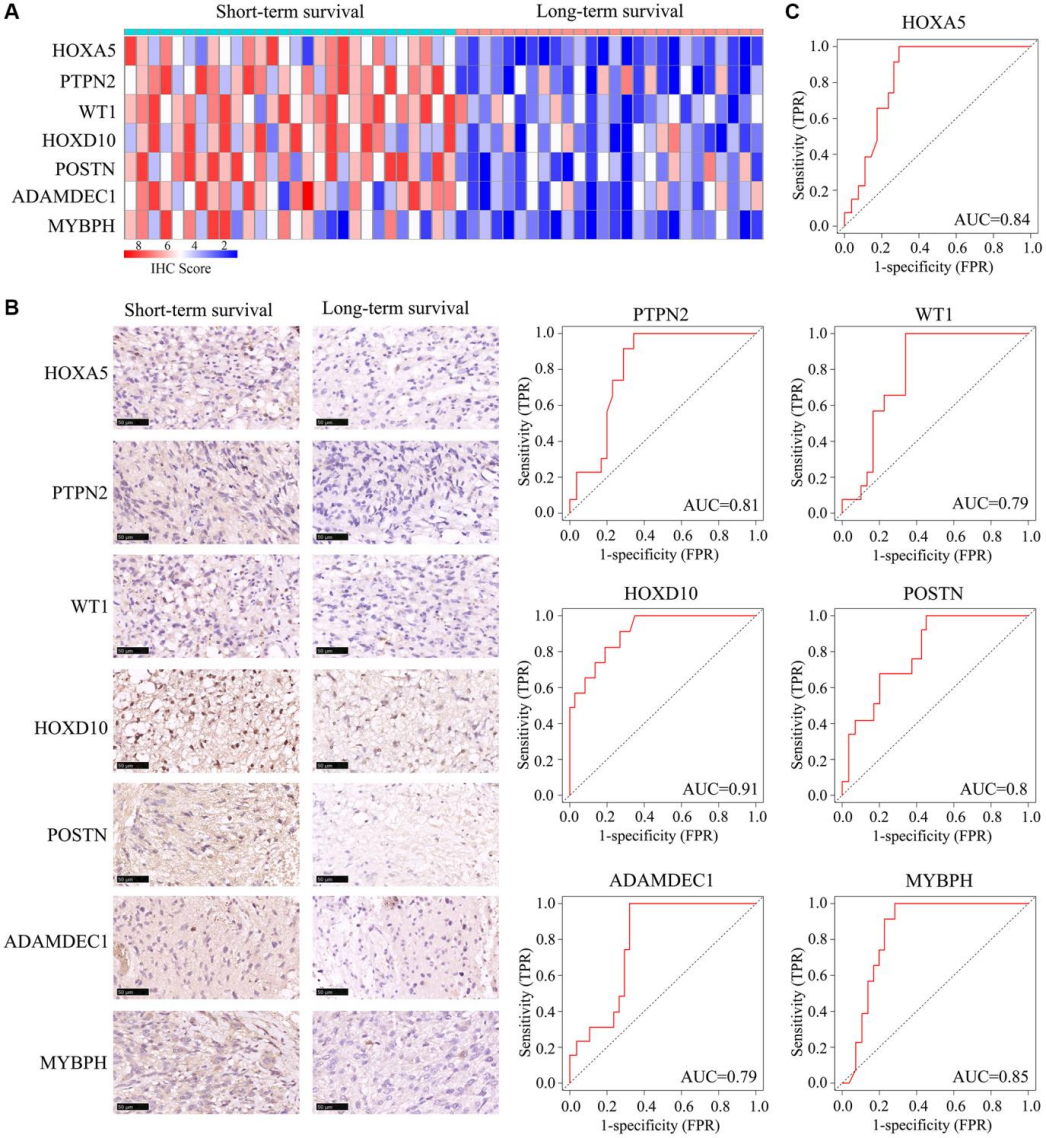
**Expression of *HOXA5, PTPN2, WT1, HOXD10, POSTN, ADAMDEC1* and *MYBPH* in *ATRX*-wt glioma tissues.*** ATRX*-wt patients were divided into long-term and short-term survival groups based on a cut-off of 15 months. (**A**) Immunohistochemistry scores for *HOXA5, PTPN2, WT1, HOXD10, POSTN, ADAMDEC1* and *MYBPH* in *ATRX*-wt glioma tissues. (**B**) Representative plots of *HOXA5, PTPN2, WT1, HOXD10, POSTN, ADAMDEC1* and *MYBPH* expression in *ATRX*-wt glioma tissues from the long- and short-term survival groups. (**C**) Prognostic value of *HOXA5, PTPN2, WT1, HOXD10, POSTN, ADAMDEC1* and *MYBPH1* in distinguishing *ATRX*-wt glioma patients with long- and short-term survival.

Next, we obtained 54 glioma tissues from patients at Guizhou Medical University Affiliated Hospital, and divided them according to whether the patients survived long-term (≥15 months) or short-term (<15 months). Then, we performed quantitative real-time PCR and immunohistochemical analyses to detect the mRNA and protein levels of *HOXA5, PTPN2, WT1, HOXD10, POSTN, ADAMDEC1* and *MYBPH* in these tissues. These genes were expressed at significantly higher levels in glioma tissues from short-term survivors than in those from long-term survivors (Supplementary Figure 1; Figure 9A, 9B). A receiver operating characteristic curve analysis indicated that *HOXA5, PTPN2, WT1, HOXD10, POSTN, ADAMDEC1* and *MYBPH* had significant prognostic value for the survival of *ATRX*-wt glioma patients (AUC = 0.84, 0.81, 0.79, 0.91, 0.80, 0.79 and 0.85, respectively) (Figure 9C).

### Immunological properties of immune markers

**Figure 10:**
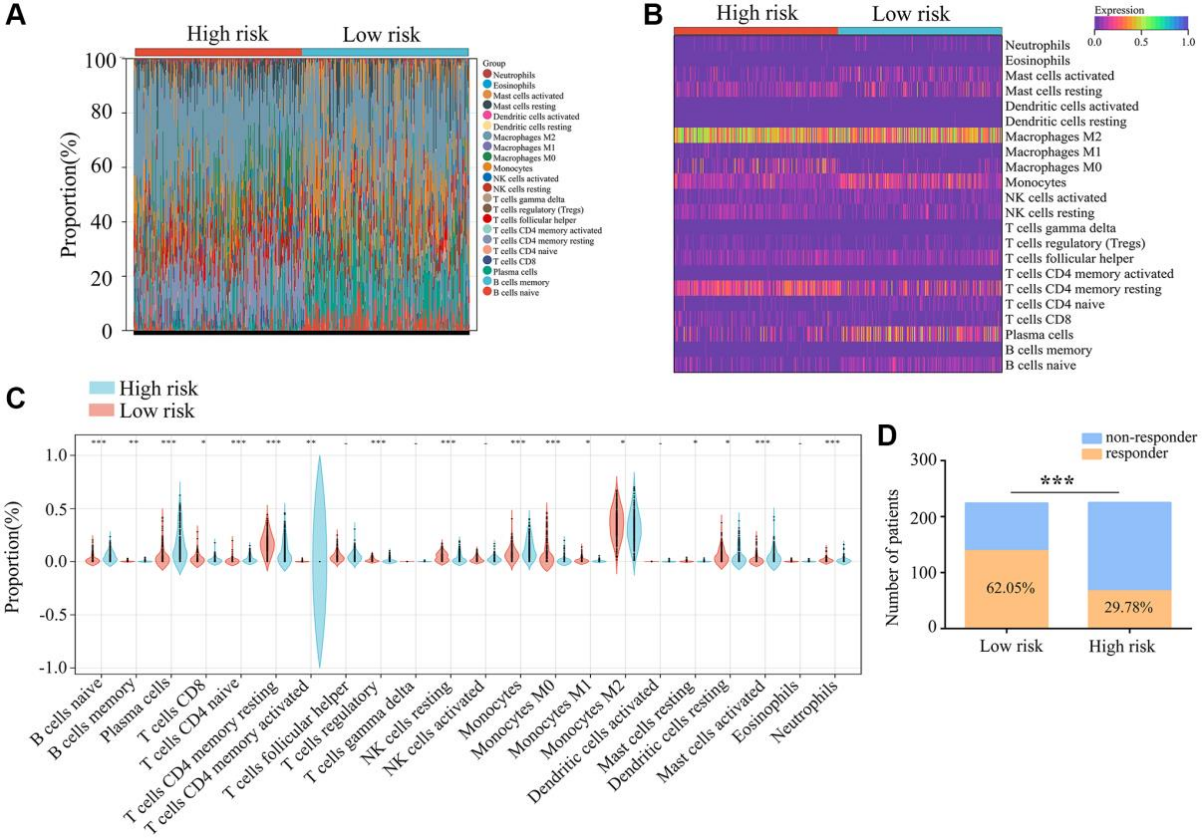
**Immunological characteristics of the three immune features.** (**A**, **B**) Gene expression profiles of the high- and low-risk-score groups of *ATRX*-wt glioma tissues from TCGA were transformed into 22 immune cell expression matrices. (**C**) Immune cell differences between the high- and low-risk groups of *ATRX*-wt glioma tissues from TCGA. (**D**) Responders and non-responders to ICB treatment among *ATRX*-wt glioma patients in the high- and low-risk groups. ^*^P < 0.05; ^**^P < 0.01; ^***^P < 0.001.

We then used the “CIBERSORT” R package to evaluate immune cell infiltration in *ATRX*-wt glioma tissues from TCGA, and found that 22 types of immune cells infiltrated differently in the high- vs. low-risk groups. CD8+ T cells, naive CD4+ T cells, monocytes, M1 macrophages and M0 macrophages were in higher proportion in glioma tissues with low-risk scores (Figure 10A–10C). We also evaluated the response to ICB treatment in the high- and low-risk *ATRX*-wt groups, and found that patients in the low-risk group had a higher response rate to ICB treatment (Figure 10D). Thus, our risk model can be used to predict the prognosis and treatment responsiveness of *ATRX*-wt patients.

### Patients with high-risk glioma may benefit from rapamycin, dasatinib, 5-fluorouracil and gemcitabine

**Figure 11:**
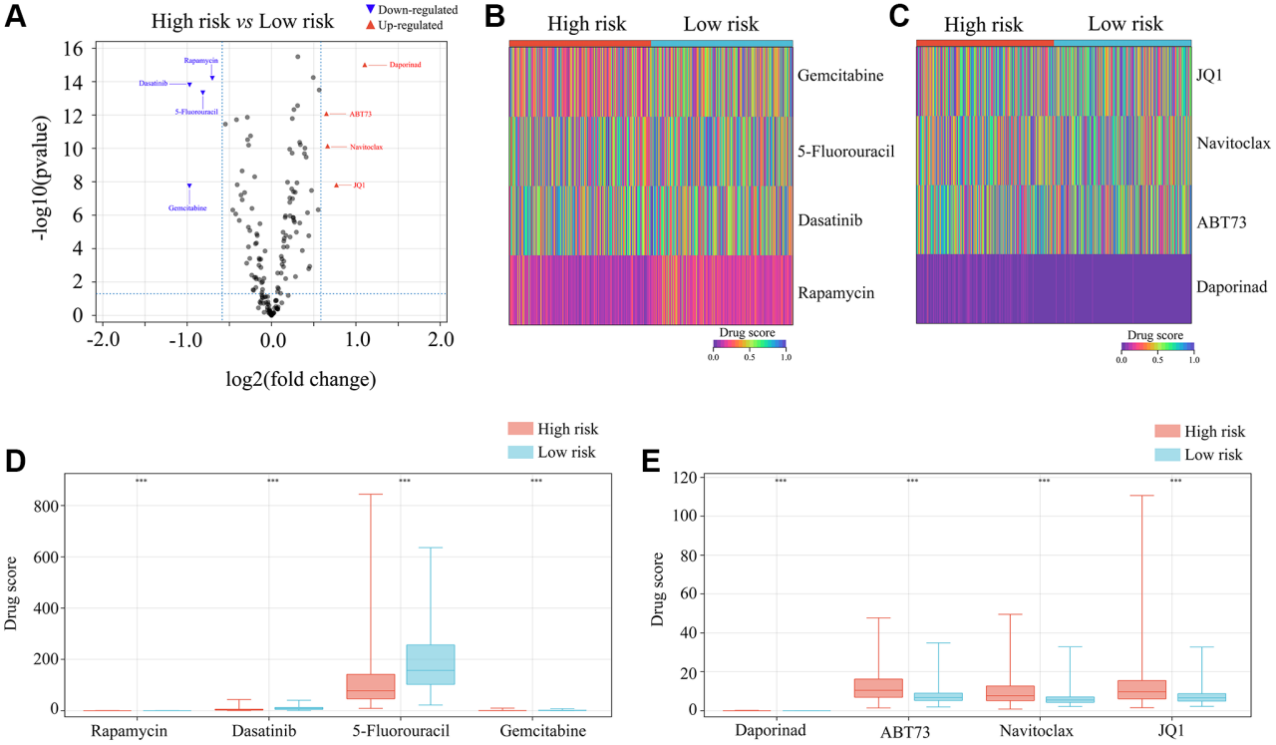
**Selection of appropriate drugs for glioma patients in the high-risk group.** (**A**–**E**) OncoPredict showed that the drug scores for rapamycin, dasatinib, 5-fluorouracil and gemcitabine differed between glioma patients in the high- and low-risk groups. ^***^P < 0.001.

Lastly, we used the OncoPredict algorithm to derive a drug sensitivity model from the gene expression data in the *ATRX*-wt atlas. We evaluated a total of 198 inhibitors, and noted that high-risk glioma tissues were predicted to be sensitive to rapamycin, dasatinib, 5-fluorouracil and gemcitabine (Supplementary Table 3; Figure 11A–11E). This evidence suggests that rapamycin, dasatinib, 5-fluorouracil and gemcitabine may be useful drugs for patients with high-risk gliomas.

## DISCUSSION

Abnormal *ATRX* expression has been detected in a variety of malignant tumors [10, 11]. *ATRX* mutations have been observed in neuroblastomas, pancreatic neuroendocrine tumors and pediatric osteosarcomas [12, 13]. *ATRX* protein contains a C-terminal helicase/ATPase domain, and belongs to the SWI/SNF2 chromatin remodeling protein family [14]. The N-terminal *ATRX–*DNA methyltransferase 3*–*DNA methyltransferase 3-like (‘ADD’) domain contains a plant homeodomain and a GATA zinc finger structure [15]. The GATA zinc finger can bind to DNA or chromatin, while the plant homeodomain participates in chromatin regulation and transcription [16, 17].

Previous studies have indicated that the immune microenvironment influences the progression of *ATRX*-wt gliomas [12]. We compared the survival of *ATRX*-wt glioma patients with high and low stromal/immune scores, and found that patients with low scores had a higher survival rate. We also assessed the DEGs between *ATRX*-wt glioma patients with high and low stromal/immune scores, and used them to generate a PPI network. We found that 80 of the 162 DEGs interacted with other genes and were significantly associated with patients’ prognoses. Using LASSO and Cox regression analyses, we identified *HOXA5*, *PTPN2*, *WT1*, *HOXD10*, *POSTN*, *ADAMDEC1* and *MYBPH* as independent predictors of survival. Based on the expression of these genes, we then generated immune profiles to classify *ATRX*-wt glioma patients as high- or low-risk.

The seven genes identified in this study have been associated with cancer in previous studies. For instance, alterations in the HOX family members *HOXA5* and *HOXD10* have been implicated in the development and progression of cancer [18, 19]. In non-small cell lung cancer, *HOXA5* was found to promote apoptosis and inhibit proliferation by upregulating linc00312 expression [20]. *HOXD10* was identified as a biological correlate of tumor suppressor DNA and an inducer of miRNA-7 and insulin-like growth factor binding protein 3 in colorectal cancer [21]. In tumor cells, *PTPN2* was shown to enhance antigen presentation and growth arrest [22]. *WT1* has been detected in hematologic malignancies and solid tumors (breast, lung, pancreatic and prostate cancers). Furthermore, *WT1* protein has high immunogenicity, suggesting that it may be a useful therapeutic agent in patients with *WT1* gene amplification [23]. Ovarian cancer cells incorporating *POSTN* from cancer-associated fibroblasts were reported to migrate and invade more effectively due to phosphoinositide 3-kinase/Akt pathway activation [24]. In the same study, the pro-metastatic and fibroblast-activating properties of transforming growth factor β1 were shown to depend partially on *POSTN* [24]. *MYBPH* was found to suppress Rho-associated coiled-coil containing protein kinase 1 and inhibit actin organization, thus impairing single cell motility, increasing collective cell migration, and reducing cancer invasion and metastasis [25]. Downregulation of *ADAMDEC1* was shown to upregulate active caspases 3 and 9, inhibit proliferation and induce apoptosis in glioma cells [26]. We constructed a risk model using *HOXA5*, *PTPN2*, *WT1*, *HOXD10*, *POSTN*, *ADAMDEC1* and *MYBPH* expression data from a cohort of patients in TCGA, and found that it had significant prognostic value for *ATRX*-wt glioma patients.

The tumor environment consists of cancer cells, immune cells, inflammatory cells, tumor-associated fibroblasts and various cytokines [27]. Immune cells within the tumor microenvironment influence the progression of glioma [27]. Glioma tissues contain a high proportion of M2 macrophages, which have the potential to promote glioma cell invasion through angiogenesis, while M1 cells have the potential to suppress angiogenesis [28, 29]. Natural killer cells and CD8+ T cells are susceptible to senescence in gliomas [30, 31]. Little has been known about the immune signature of *ATRX*-wt glioma, but we found that *ATRX*-wt patients with low risk scores tended to have higher levels of M1 and CD8+ T cells, suggesting that these infiltrating cells were able to kill cancer cells.

In order for the host to kill cancer cells, immune checkpoints need to be blocked so that deactivated cells can be reactivated. ICB therapy has shown significant curative effects in hepatocellular carcinoma and breast cancer patients [32, 33]. We found evidence that CD8+ T cell and M1 cell inactivation were inhibited in the tumor microenvironment of *ATRX*-wt glioma patients with low risk scores; thus, we analyzed whether ICB therapy was beneficial for low-risk *ATRX*-wt glioma patients. ICB therapy response rates were higher among low-risk patients than among high-risk patients, suggesting that *ATRX*-wt glioma patients with low risk scores may benefit from ICB therapy. An OncoPredict analysis predicted that high-risk glioma patients would be more sensitive to rapamycin, dasatinib, 5-fluorouracil and gemcitabine.

In conclusion, our study revealed that an immune signature based on *HOXA5*, *PTPN2*, *WT1*, *HOXD10*, *POSTN*, *ADAMDEC1* and *MYBPH* expression effectively predicted the prognosis of *ATRX*-wt glioma patients, and demonstrated that immunotherapy was effective for low-risk patients. Our immune signature may be helpful in diagnosing and treating *ATRX*-wt glioma patients.

## MATERIALS AND METHODS

### Downloading and preprocessing gene expression profiles

The gene expression profiles and clinical characteristics of 452 *ATRX*-wt and 188 *ATRX*-mt glioma patients were obtained from TCGA. The gene expression profiles were normalized and centralized, and gene names were assigned to the probes. The immune and stromal scores of the *ATRX*-wt glioma tissues were calculated using the ESTIMATE algorithm.

### DEG analysis

The median immune and stromal scores from the dataset in TCGA were used to divide patients into high- or low-scoring groups. DEG analysis was performed using EdgeR, with an adjusted *P*-value of 0.05 and a |logFold-Change| <1 set as the threshold for significance. Volcano plots and heat maps were used to visualize gene expression changes in the groups with high immune/stromal scores [34].

### PPI network

A PPI network was constructed by mapping DEG information to the Search Tool for the Retrieval of Interacting Genes (STRING) database. Isolated genes were removed from the original PPI network using Cytoscape software. A visual analysis was performed, and reciprocally related genes were designated as hub genes and included in the next step.

### Functional and pathway enrichment analysis

The Database for Annotation, Visualization and Integrated Discovery was used to analyze the enriched KEGG and Gene Ontology terms of genes. Three categories were used for the Gene Ontology analysis: Biological Processes, Cellular Components and Molecular Functions. A bubble diagram was used to present the terms to determine significance.

### Immune signature construction and verification

In *ATRX*-wt patients, a univariate Cox regression analysis with a significance threshold was used to construct an immune signature. A LASSO operator with an appropriate penalty was used to eliminate genes with the same genetic information. A prognostic risk model was developed using multivariate Cox regression analysis with the Akaike information criterion. Kaplan-Meier survival analyses and receiver operating characteristic curve analyses were used for *ATRX*-wt and *ATRX*-mt glioma patients [35].

### Construction of column line diagrams

The “rms” package in R was used to create column line plots and conduct a multi-factor regression analysis. Asymptotic lines were then used to plot on the same plane at a certain scale. The accuracy of the line plot was predicted, and the prognostic value of the line plot was determined.

### Immune cell analysis

Using the “CIBERSORT” R package, we examined 22 immune cells infiltrating *ATRX*-wt glioma tissues in TCGA. Differentially infiltrated cells in the high- and low-risk-score groups were analyzed using the unpaired *t*-test, with significance set as *P* < 0.05.

### Immunohistochemical analysis

Throughout Guizhou Medical University Affiliated Second People Hospital of Guiyang, 54 *ATRX*-wt glioma tissues were collected with approval from the Guizhou Medical University Human Ethics Committee. All participants provided informed consent before they were given radiotherapy or chemotherapy. Immunohistochemical staining was performed as described in a previous study [36]. The sections were probed with the following antibodies: *HOXA5* (1:200; ab140636, Abcam, Cambridge, UK), *PTPN2* (1:100; 11214-1-AP, Proteintech, Wuhan, China), *WT1* (1:100; 12609-1-AP, Proteintech), *POSTN* (1:100; 66491-1-Ig, Proteintech), *ADAMDEC1* (1:100; 17899-1-AP, Proteintech), *MYBPH* (1:100; ab197216, Abcam) and *HOXD10* (1:100; ab138508, Abcam).

### Drug score analysis

*In vivo* drug responses can be predicted using OncoPredict, an algorithm developed by Maeser et al. [37]. In order to calculate the drug sensitivity of gliomas, OncoPredict scripts were used to match the gene expression matrix of each glioma sample to the chemotherapeutic effects of drugs recorded in Cancer and the gene expression information for cancer lines in the Broad Institute Cancer Cell Line Encyclopedia. Glioma patients with higher drug scores are less sensitive to drugs. The limma package was used to analyze the differences in drug scores between those at high and low risk. A |logFold-Change| ≥1 and adjusted *P* < 0.05 were used as cut-offs for determining significance.

### Availability of data and materials

The datasets used and/or analyzed during the current study are available from the corresponding author on reasonable request.

## Supplementary Materials

Supplementary Figure 1

Supplementary Table 1

Supplementary Table 2

Supplementary Table 3
