# Supplementary material for: An immune signature to predict the prognosis of ATRX-wildtype glioma patients and guide immune checkpoint blockade therapy
Source: Aging (Albany NY). 2023 Oct 6;15(19):10453–72. doi: 10.18632/aging.205088 (PMC10599764; doi:10.18632/aging.205088)
Supplement: Supplementary Figure 1 [file aging-15-205088-s001.pdf]

SUPPLEMENTARY FIGURE

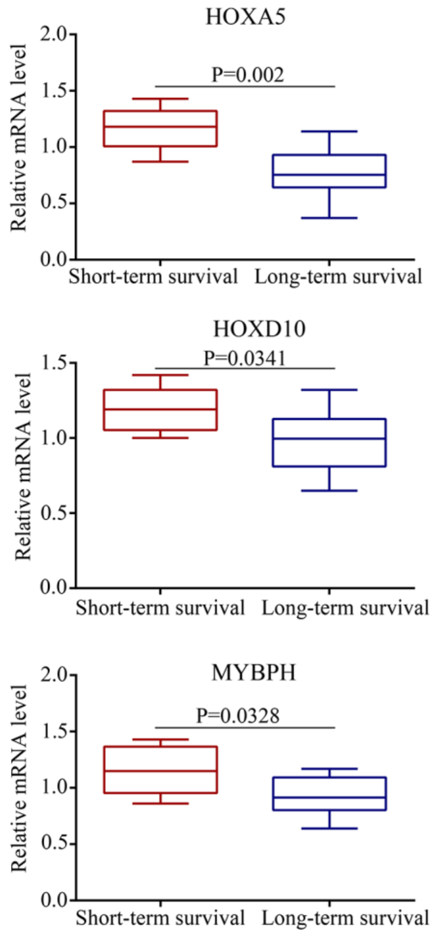

Supplementary Figure 1. The mRNA levels of *HOXA5*, *PTPN2*, *WT1*, *HOXD10*, *POSTN*, *ADAMDEC1* and *MYBPH* in each subtype of glioma.
